# First-Principles Investigation of Size Effects on Cohesive Energies of Transition-Metal Nanoclusters

**DOI:** 10.3390/nano13162356

**Published:** 2023-08-17

**Authors:** Amogh Vig, Ethan Doan, Kesong Yang

**Affiliations:** 1 Department of Nano and Chemical Engineering, University of California San Diego, 9500 Gilman Drive, Mail Code 0448, La Jolla, CA 92093-0448, USA; amogh.vig@vanderbilt.edu (A.V.); ekdoan@ucsd.edu (E.D.); 2 Data Science Institute, Vanderbilt University, 2201 West End Ave., Nashville, TN 37325-0001, USA

**Keywords:** nanocluster, cohesive energy, transition-metal, DFT, first-principles

## Abstract

The cohesive energy of transition-metal nanoparticles is crucial to understanding their stability and fundamental properties, which are essential for developing new technologies and applications in fields such as catalysis, electronics, energy storage, and biomedical engineering. In this study, we systematically investigate the size-dependent cohesive energies of all the 3*d*, 4*d*, and 5*d* transition-metal nanoclusters (small nanoparticles) based on a plane-wave-based method within general gradient approximation using first-principles density functional theory calculations. Our results show that the cohesive energies of nanoclusters decrease with decreasing size due to the increased surface-to-volume ratio and quantum confinement effects. A comparison of nanoclusters with different geometries reveals that the cohesive energy decreases as the number of nanocluster layers decreases. Notably, monolayer nanoclusters exhibit the lowest cohesive energies. We also find that the size-dependent cohesive energy trends are different for different transition metals, with some metals exhibiting stronger size effects than others. Our findings provide insights into the fundamental properties of transition-metal nanoclusters and have potential implications for their applications in various fields, such as catalysis, electronics, and biomedical engineering.

## 1. Introduction

Nanoparticles have garnered considerable attention as promising tools for technological advancements across diverse disciplines [1,2,3,4,5,6,7,8,9,10,11]. Their distinct optical, electronic, magnetic, and reactive properties set them apart from bulk transition-metal structures [1,2]. This unique attribute has opened a wide range of potential applications, including drug delivery, therapeutics, environmental remediation, and energy storage, among others [2,3,4,5,6]. Of particular significance for our research focus, transition-metal nanoparticles have emerged as compelling candidates for catalytic applications in crucial industrial processes, such as methane reformation [12,13]. The catalytic potential of these nanoparticles holds great promise for enhancing efficiency and sustainability in various industries. 

One of the most important physical properties of transition-metal nanoparticles is their cohesive energy, which is defined as the energy required to fully decompose a nanoparticle into its constituent atoms [14,15]. Cohesive energy is strongly correlated to a wide range of thermo-physical properties, which include, but are not limited to, melting temperature, melting enthalpy, and the creation and diffusion of vacancies of nanomaterials [16,17,18,19]. For instance, in the realm of catalysis, cohesive energy plays a crucial role in determining the stability and robustness of transition-metal nanoparticles as catalysts [2,11,20]. Higher cohesive energy values signify greater stability, a crucial characteristic for successful catalysts that need to lower activation energy and accelerate reactions without self-destruction. Moreover, it is essential to acknowledge that the cohesive energy of transition-metal nanoparticles is influenced by their structural configuration and total number of atoms, making it vital to comprehend and predict cohesive energy trends across different nanoparticle sizes and configurations, especially for screening potential catalytic candidates. 

Therefore, understanding and predicting the cohesive energy trends of transition-metal nanoparticles across different nanoparticle sizes and configurations is of vital importance in optimizing their performance and designing tailored materials for catalytic advancements. Prior studies have been carried out to understand the size and structural dependency of cohesive energy for a select group of transition-metal nanoparticles such as Ag, Al, W, Co, Mo, Pt, Cu, and Au [16,17,18]. These studies, however, mostly relied on empirical methods for calculating cohesive energy, such as the bond energy model (BEM) [16] and other simple models [17,18,19]. As a comparison, a first-principles approach was employed to investigate various aspects of nanomaterials in previous studies. Specifically, it was used to study the geometries and stabilities of bimetallic nanoparticles, such as Fe-Ni nanoparticles [21] and doped Au clusters [22]. Additionally, this approach was utilized to explore the catalytic mechanism of specific nanoclusters, such as Ni_4_ [23] or three-atom metal clusters [24]. Moreover, the thermal stability of nanoparticles, which varies based on their sizes [16,17,18], shapes [10], and surrounding environment [9], continues to be an active research topic awaiting further elucidation. 

In this work, we conducted first-principles density functional theory (DFT) calculations to determine the size-dependent cohesive energies of all the 3*d*, 4*d*, and 5*d* transition-metal nanoclusters (small nanoparticles) with one-layer, two-layer, and three-layer geometries. This comprehensive investigation allowed us to unveil strong and applicable trends across diverse chemical species, nanocluster sizes, and structures. By gaining these valuable insights, we can better evaluate and predict cohesive energy in transition-metal nanoclusters, especially those with potential significance in industrial catalysis. Our findings hold the potential to guide the selection of optimal transition-metal nanocluster candidates for catalytic processes, ultimately contributing to advancements in various industrial applications.

## 2. Computational Details

First-principles DFT electronic structure calculations were performed using the Vienna ab initio simulation package (VASP) [25,26]. The projector augmented wave potentials (PAW) were used for treating electron–ion interactions, and the generalized gradient approximation parametrized by Perdew–Burke–Ernzerhof was used for exchange–correlation functions [27,28]. Structures were fully relaxed with a convergence tolerance of 0.01 meV per atom, and a single *k*-point with the wavevector at the Γ point was used in our calculations. Other computational settings such as cut-off energy and appropriate entries for structural relaxations were generated and managed by the high-throughput computational software framework AFLOW code [29]. For instance, the maximum cutoff energy from the pseudopotential files was automatically chosen for all our calculations. 

**Figure 1:**
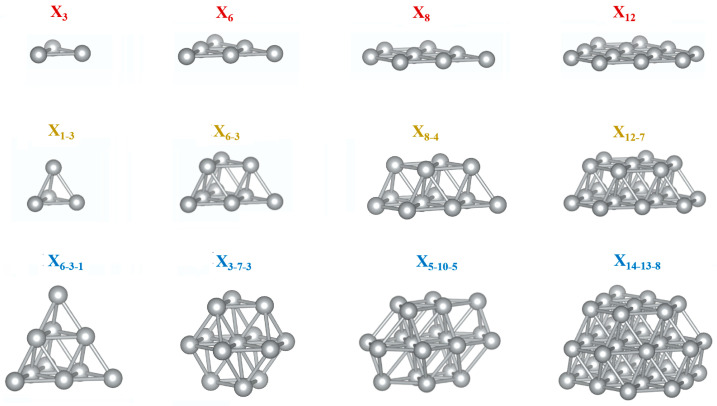
Illustration of geometric structures of transition-metal (X) nanoclusters, with the first, second, and third rows corresponding to single-layer, two-layer, and three-layer nanoclusters, respectively.

An illustration of the geometrical structures of all the studied transition-metal nanoclusters (X*_n_*, X = 3*d*, 4*d*, and 5*d* transition-metal elements) is shown in Figure 1. These structures were built based on a prior computational study of Pt nanoparticles conducted by Brunello et al. [30]. These nanocluster structures include one-layer, two-layer, and three-layer cluster configurations, totaling 12 structures, each showcasing distinct arrangements of X atoms within the nanoclusters. This leads to a total number of 360 configurations for all the studied transition-metal nanoclusters. Through these configurations, insights into the nanoclusters’ stability and properties at different sizes can be obtained. These nanoparticle models are placed in a cubic simulation box to mimic the bulk environment. To avoid interactions between periodic images, a vacuum region with a minimum distance of at least 10 Å is introduced between neighboring images of the nanoclusters. This ensures that the nanoclusters do not interact with their periodic replicas in the simulation, preventing artificial effects and providing an accurate representation of the isolated nanoclusters. Our benchmark calculations show that a separation distance of 10 Å between adjacent images of the nanoclusters is sufficient to model the isolated nanoparticles (see Figure S1 of Supplementary Information). 

## 3. Results and Discussion

Cohesive energy refers to the energy needed to fully disassemble the constituent atoms of a substance, specifically in the case of a nanocluster, and separate them from each other [14,15]. Consequently, the cohesive energy of transition-metal nanocluster structures was determined through a two-step calculation process. First, the total energy of the entire nanoparticle system was obtained. This energy accounts for the interactions and bonding between atoms within the nanoparticle. Next, the energy of an individual metal atom (*E_atom_*) was calculated separately. This energy represents the energy of an isolated metal atom. Finally, the cohesive energy per atom (*E_coh_*) was calculated by subtracting the energy of the individual atom from the total energy per atom of the nanoparticle system: *E_coh_* = *E_atom_* − *E_tot_/n*, where *E_tot_* is the total energy of the nanoparticle structure and *n* is the number of atoms of the structure.

To succinctly depict the relationship between cohesive energy and nanoparticle size, we employed a natural logarithmic function for their approximation, as shown below:

$$Y=a\log_{e}\left(bn\right)=a\ln\left(bn\right)$$

**Figure 2:**
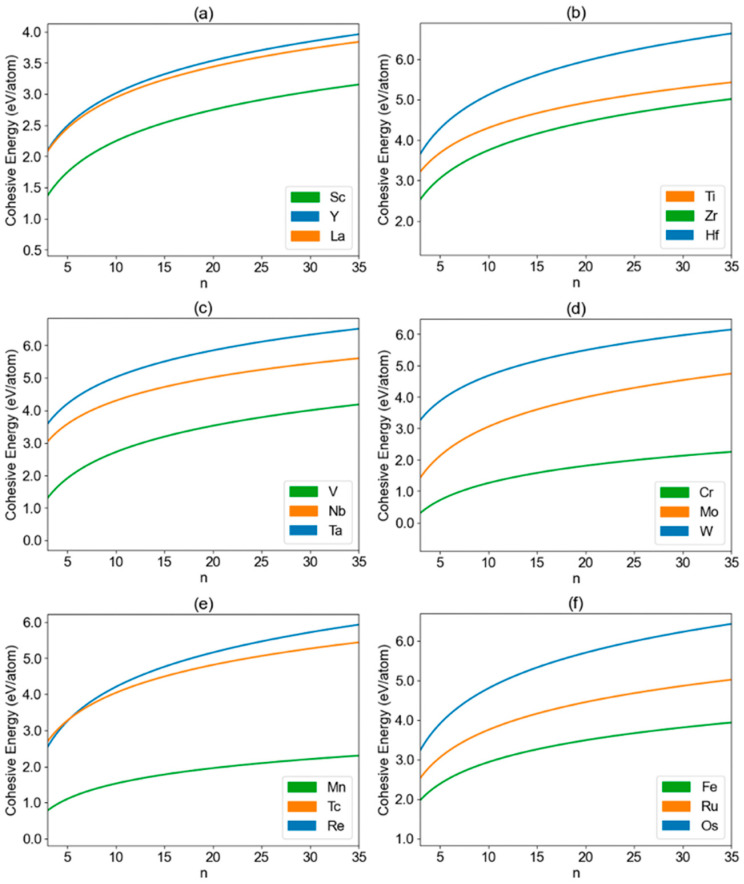
Calculated cohesive energy per atom as a function of the nanocluster size (*n*) for the transition-metal nanoparticles (X*_n_*) in the group (**a**) 3B (X = Sc, Y, and La), (**b**) 4B (X = Ti, Zr, and Hf), (**c**) 5B (X = V, Nb, and Ta), (**d**) 6B (X = Cr, Mo, and W), (**e**) 7B (X = Mn, Tc, and Re), and (**f**) 8B (x = Fe, Ru, Os). The size *n* refers to the number of atoms of the nanocluster.

**Figure 3:**
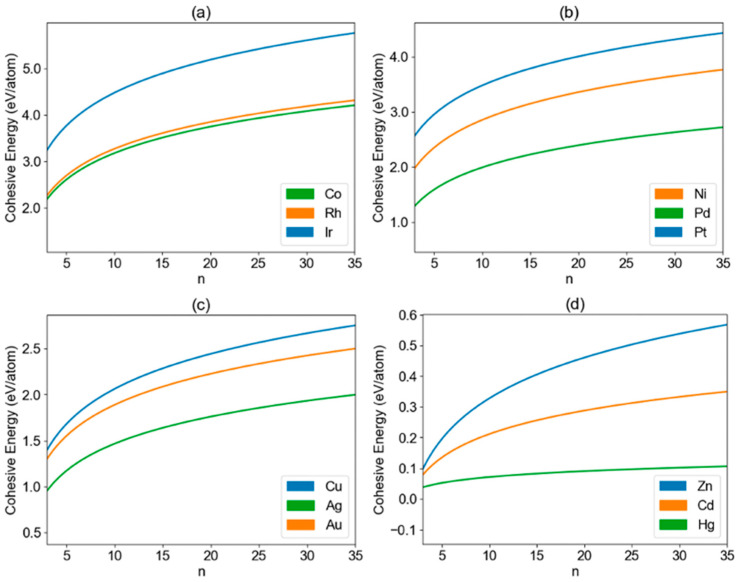
Calculated cohesive energy per atom as a function of the nanocluster size (*n*) for the transition-metal nanoclusters (X*_n_*) in the group (**a**) 8B (X = Co, Rh, and Ir), (**b**) 8B (X = Ni, Pd, and Pt), (**c**) 1B (X = Cu, Ag, and Au), and (**d**) 2B (X = Zn, Cd, and Hg).

In this logarithmic function, *Y* represents the cohesive energy per atom, *n* denotes the nanoparticle size, and *a* and *b* are parameters unique to each transition-metal element. A list of specific parameters, *a* and *b*, for all the elements studied in this research is summarized in Table S1 of the Supplementary Information. The calculated cohesive energy for all the transition-metal nanoclusters is summarized in Tables S2–S11 of the Supplementary Information. In Figure 2, the plotted curve represents the fitted cohesive energy of transition-metal nanoclusters (X*_n_*) as a function of nanoparticle size (*n*) for groups 3B to 8B. The curve is obtained through the natural logarithmic fitting, providing a concise representation of the cohesive energy trends within these groups. Here, the nanoparticle size (*n*) represents the number of atoms in the nanoparticle. Similarly, Figure 3 presents the fitted cohesive energy results for the groups 8B (X = Co, Rh, and Ir; X = Ni, Pd, and Pt), 1B (X = Cu, Ag, and Au), and 2B (X = Zn, Cd, and Hg). The calculated specific cohesive energy of each nanoparticle structure along with the fitted curve for all the studied transition-metal nanoclusters are shown in Figures S2–S7 of the Supplementary Information.

For all the groups, cohesive energy per atom increases with the size (*n*) of the nanoclusters, eventually approaching the values observed in the bulk material. This trend indicates a convergence towards the cohesive behavior characteristic of the corresponding bulk materials. This size-dependent increment in cohesive energy is attributed to the strengthening of interatomic interactions and enhanced bonding as the nanoparticle size expands. Specifically, as nanoclusters diminish in size, the decrease in cohesive energy can be attributed to the amplified surface-to-volume ratio and quantum confinement effects. A similar trend was also observed in a very recent study conducted by Sachin et al. [16], in which cohesive energy analysis was carried out for elements Ag, W, Co, and Mo via the bond energy model. 

In most of the studied groups, the cohesive energy displays a considerable range, spanning from 0.50 eV per atom to 6.00 eV per atom. This wide variability in cohesive energy values exhibits strong size effects and highlights the diverse nature of these transition-metal nanoclusters and their potential for applications in various fields. However, distinctive behaviors emerge in the last two groups, namely groups 1B and 2B, where the cohesive energy ranges are notably smaller, indicating weaker size effects than other groups. The varying strength of size effects among different metals is fundamentally attributed to the intrinsic attributes of each metal species, including factors such as electronic configuration and strength of metallic bonding. For instance, elements within groups 1B and 2B typically exhibit weaker metallic bonds in comparison to other groups. For group 1B, the cohesive energy values exhibit a more constrained range, varying from approximately 1.00 eV per atom to around 2.80 eV per atom. These values are also consistent with the previously calculated average atomic binding energies of Pt-group-doped gold clusters in the range of 1.56–2.00 eV [22]. In the case of group 2B nanoclusters, the cohesive energy range is even narrower, spanning from about 0.05 eV per atom to approximately 0.58 eV per atom. This remarkable narrowing of cohesive energy values suggests unique characteristics within group 2B nanoclusters. Such nanoclusters may hold promise for specialized applications that require precise control over cohesive energy properties [31]. 

Analyzing the trends in cohesive energy per atom for individual groups yields the following observations:

(i) In group 5B (X = V, Nb, and Ta), there is a consistent increase in cohesive energy per atom with the atomic number. Ta stands out with the highest cohesive energy per atom, while V exhibits the lowest, and Nb falls in between. This upward trend is also evident in other groups, including 6B (X = Cr, Mo, and W), 7B (X = Mn, Tc, and Re), 8B (X = Fe, Ru, Os), and 8B (X = Co, Rh, and Ir), reflecting a common pattern among these transition-metal nanoclusters.

(ii) Group 4B (X = Ti, Zr, and Hf) presents a different trend. Surprisingly, the middle element, Zr, displays the lowest cohesive energy per atom, while the element with the highest atomic number, Hf, showcases the highest cohesive energy per atom. Ti, with the lowest atomic number, positions its cohesive energy per atom between that of Zr and Hf. Remarkably, this trend extends to group 8B (X = Ni, Pd, and Pt) as well, further highlighting the unique behavior observed within this group.

(iii) Another intriguing pattern emerges with group 3B (X = Sc, Y, and La). Here, the middle element, Y, exhibits the highest cohesive energy per atom, while the element with the lowest atomic number, Sc, demonstrates the lowest cohesive energy per atom. La, the element with the highest atomic number, displays cohesive energy per atom between that of Sc and Y. This behavior offers insights into the cohesive characteristics of these nanoclusters, reflecting a distinctive trend within this group.

(iv) Group 1B (X = Cu, Ag, and Au) exhibits a highly distinctive trend compared to the other groups. The element with the lowest atomic number, Cu, surprisingly demonstrates the highest cohesive energy per atom. Conversely, the middle element, Ag, displays the lowest cohesive energy per atom among the three elements. Lastly, the element with the highest atomic number, Au, showcases cohesive energy per atom between that of Cu and Ag. This exceptional behavior adds to the complexity of cohesive energy trends within transition-metal nanoclusters.

(v) The final group, 2B (X = Zn, Cd, and Hg), presents a trend opposite to that observed in point i. In this group, cohesive energy per atom decreases with the atomic number. Zn stands out with the highest cohesive energy per atom, while Hg exhibits the lowest cohesive energy, and Cd falls in between. This contrasting trend indicates the diverse cohesive energy behavior within these nanoclusters.

(vi) Lastly, when comparing different geometries of nanoclusters with the same transition-metal element, it is generally observed that cohesive energy increases with the number of layers in the nanocluster structure. Specifically, despite some overlap, single-layer nanocluster structures typically exhibit lower cohesive energy, three-layer structures demonstrate higher cohesive energy, and two-layer structures fall in between. These findings are visually presented in Figures S2–S7 of the Supplementary Information, where the calculated cohesive energy for single-layer, two-layer, and three-layer nanocluster structures is distinguished using different colors.

**Figure 4:**
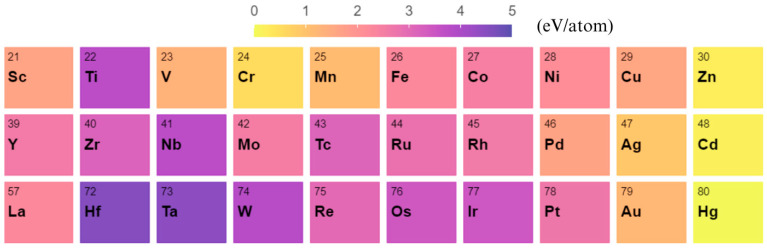
Heat map illustrating the calculated cohesive energy per atom for the smallest three-dimensional transition-metal nanoclusters X_4_.

To present a clear and comprehensive comparison of cohesive energies among all the studied transition-metal elements, we plotted a heat map illustrating the calculated cohesive energy values for the smallest three-dimensional transition-metal nanoclusters with structure X_4_ (see Figure 4). It provides valuable insights into the cohesive energy trends across the various elements studied. For instance, one can observe that Hf has the highest cohesive energy, closely followed by Ta, while the nanoclusters of group 2B (X = Zn, Cd, and Hg) have low cohesive energy, with Hg exhibiting the lowest one among all the elements studied. The overall trend of the calculated cohesive energy of the X_4_ nanocluster is generally consistent with the experimental values of the bulk transition metals in spite of some difference, as shown in Figure S7 of the Supplementary Information. For instance, the bulk compound with the highest experimental cohesive energy values is W instead of Hf. Additionally, the cohesive energy values of Cr, Mo, Re, and Os are also higher than that of their nanoparticle counterparts. In short, our calculated results and the comparison with experimental bulk values provide a concise overview of the differences in cohesive energy values for both nanoclusters and bulk structures, shedding light on the unique properties exhibited by nanoclusters compared to their bulk counterparts. 

When comparing our calculated results with the experimental values, we find that the cohesive energy values obtained in our study are usually smaller than the experimental values [32]. For instance, the experimentally determined cohesive energy for W nanoparticle is about 6.14 eV/atom for 6nm structure [32]. In contrast, the calculated highest cohesive energy obtained for W is 6.05 eV/atom in the present study. This is an unsurprising finding since most of the nanoparticle structures studied in prior works are larger than the nanoclusters that we investigated, and therefore exhibit higher cohesive energy. An interesting anomaly arises in the case of the Mo nanocluster: its experimentally determined cohesive energy value stands at approximately 4.25 eV/atom, which marginally trails our computed value of about 4.37 eV/atom [32].

## 4. Conclusions

In conclusion, we systematically explored the size-dependent cohesive energies of 3*d*, 4*d*, and 5*d* transition-metal nanoclusters using first-principles density functional theory calculations. Our investigation revealed a consistent trend of decreasing cohesive energies with decreasing nanocluster size, which can be attributed to the increased surface-to-volume ratio and quantum confinement effects. It is also found that the number of nanocluster layers decreases, the cohesive energy diminishes, with monolayer nanoclusters exhibiting the lowest cohesive energies. Importantly, we observed variations in the size-dependent cohesive energy trends among different transition metals, with certain metals exhibiting more pronounced size effects than others. These findings offer valuable insights into the fundamental properties of transition-metal nanoclusters and have implications for their applications in fields such as catalysis, electronics, energy storage, and biomedical engineering. 

## Data Availability

The data that support the findings of this study are available in the Supplementary information document. The structural files of the relaxed 360 nanocluster structures are provided online at https://github.com/ksyang2013/nanoclusters. Other relevant data are available from the corresponding author upon reasonable request.

## Supplementary Materials

The following supporting information can be downloaded at: https://www.mdpi.com/article/10.3390/nano13162356/s1, Figure S1: calculated total energy as a function of the distance between two neighboring images of Ni4 nanocluster; Figures S2–S7: calculated cohesive energy as a function of the nanocluster size (*n*) for transition-metal nanoclusters (X*_n_*) in period 4 (X = Sc, Ti, V, Cr, Mn, and Fe in Figure S2; X = Co, Ni, Cu, and Zn in Figure S3), period 5 (X = Y, Zr, Nb, Mo, Tc, and Ru in Figure S4; X = Rh, Pd, Ag, and Cd in Figure S5), and period 6 (X = La, Hf, Ta, W, Re, and Os in Figure S6; X = Ir, Pt, Au, and Hg in Figure S7); Figure S8: heat map illustrating the calculated cohesive energy for nanoclusters X_4_ and experimental values of the bulk structures; Table S1: list of parameters *a* and *b* in the natural logarithmic function for fitting cohesive energies; Tables S2–S11: calculated cohesive energy of the transition-metal nanoclusters (X*_n_*) in the group 3B (Table S2), group 4B (Table S3), group 5B (Table S4), group 6B (Table S5), group 7B (Table S6), group 8B (X = Fe, Ru, and Os in Table S7), group 8B (X = Co, Rh, and Ir in Table S8), group 8B (X = Ni, Pd, and Pt in Table S9), group 1B (Table S10), and group 2B (Table S11).
