# Supplementary material for: First-Principles Investigation of Size Effects on Cohesive Energies of Transition-Metal Nanoclusters
Source: Nanomaterials (Basel). 2023 Aug 17;13(16):2356. doi: 10.3390/nano13162356 (PMC10458230; doi:10.3390/nano13162356)
Supplement: Supplementary file 1 [file nanomaterials-13-02356-s001.zip › nanomaterials-2562916-supplementary.pdf]

Supplementary Information

First-Principles Investigation of Size Effects on Cohesive Energies of Transition-Metal Nanoclusters

Amogh Vig [1,2], Ethan Doan [1] and Kesong Yang [1,*]

[1]Department of Nano and Chemical Engineering, University of California San Diego, 9500 Gilman Drive, Mail Code 0448, La Jolla, CA92093-0448, United States

[2]Data Science Institute, Vanderbilt University, 2201 West End Ave, Nashville, TN 37325-0001, United States

*Email: kesong@ucsd.edu, Phone: +1-858-534-2514

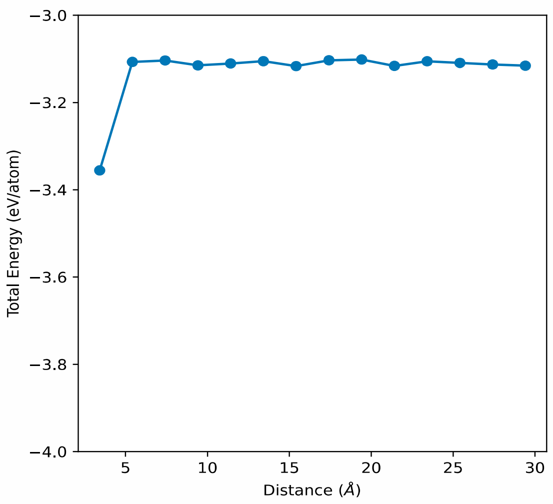

Figure S1: Calculated total energy as a function of the distance between two neighboring images of Ni_4 nanocluster.

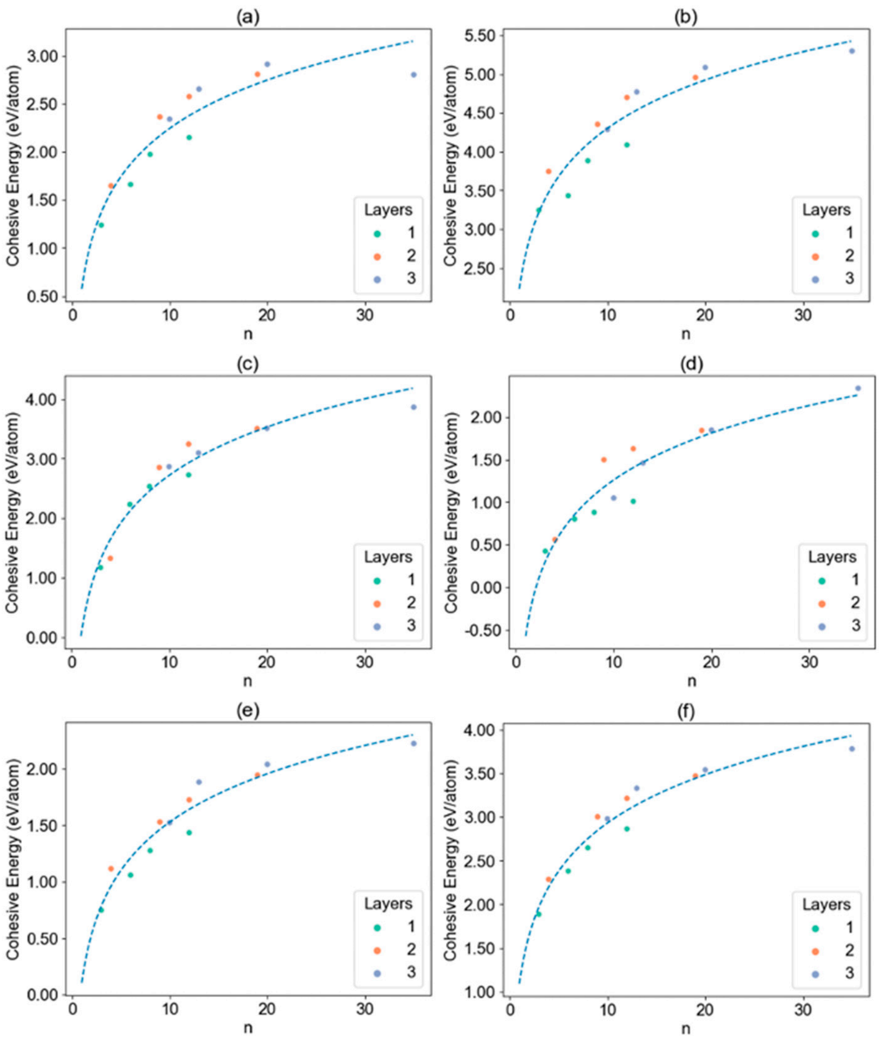

Figure S2: Calculated cohesive energy as a function of the nanocluster size (n) for transition-metal nanoclusters (X_n) in period 4 (a) X = Sc, (b) X = Ti, (c) X = V, (d) X = Cr, (e) X = Mn, and (f) X = Fe.

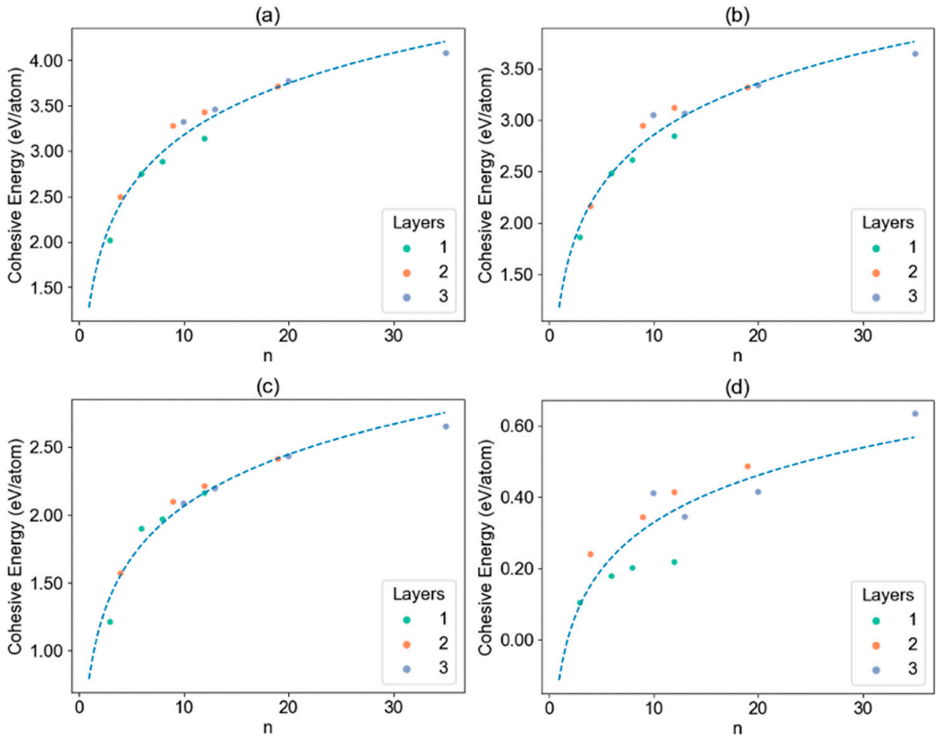

Figure S3: Calculated cohesive energy as a function of the nanocluster size (n) for transition-metal nanoclusters (X_n) in period 4 (a) X = Co, (b) X = Ni, (c) X = Cu, and (d) X = Zn.

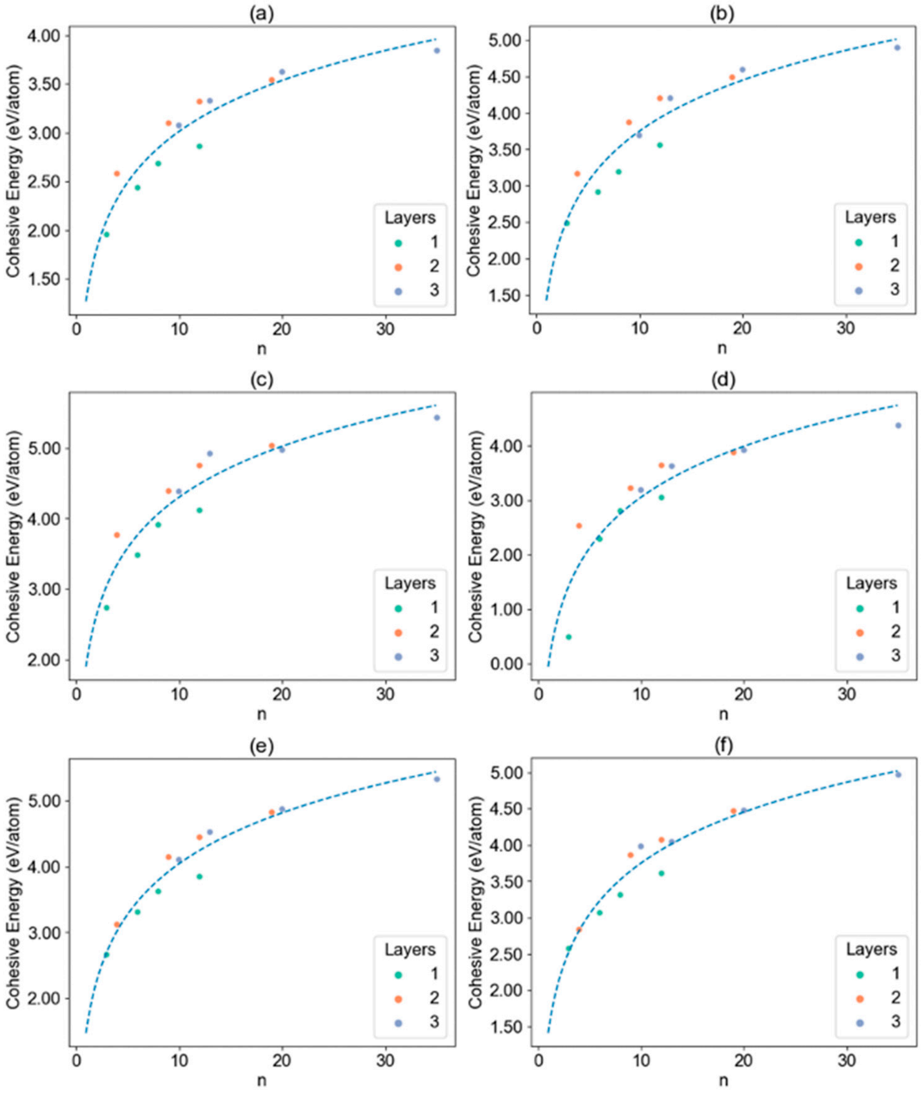

Figure S4: Calculated cohesive energy as a function of the nanocluster size (n) for transition-metal nanoclusters (X_n) in period 5 (a) X = Y, (b) X = Zr, (c) X = Nb, (d) X = Mo, (e) X = Tc, and (f) X = Ru.

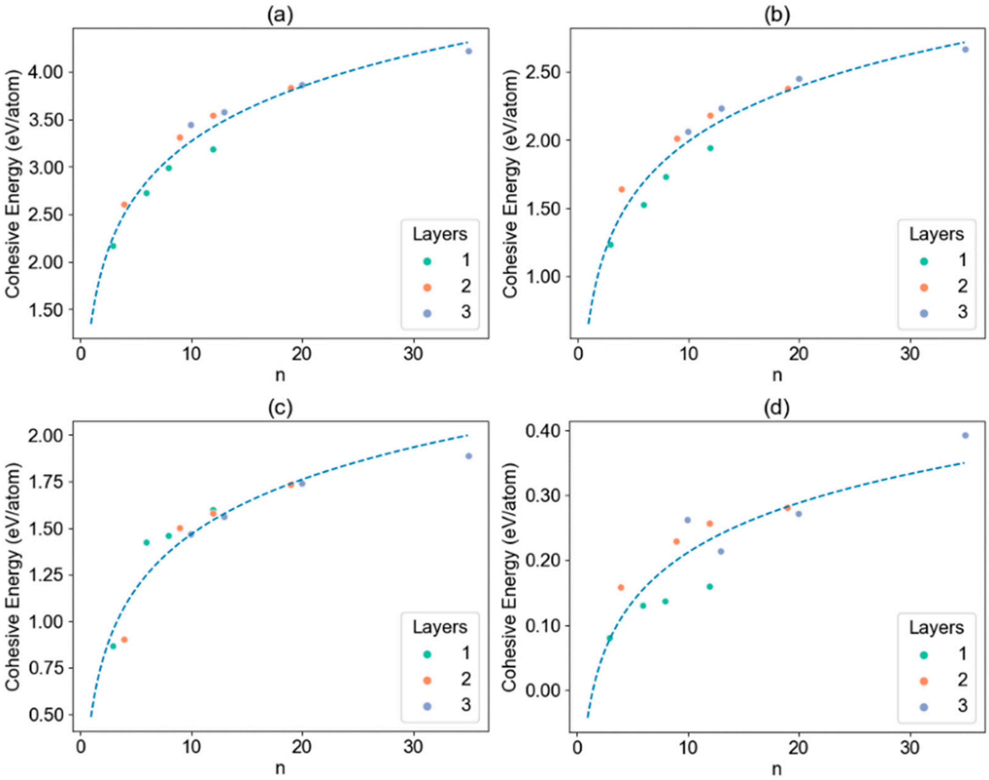

Figure S5: Calculated cohesive energy as a function of the nanocluster size (*n*) for transition-metal nanoclusters (X_n) in period 5 (a) X = Rh, (b) X = Pd, (c) X = Ag, and (d) X = Cd.

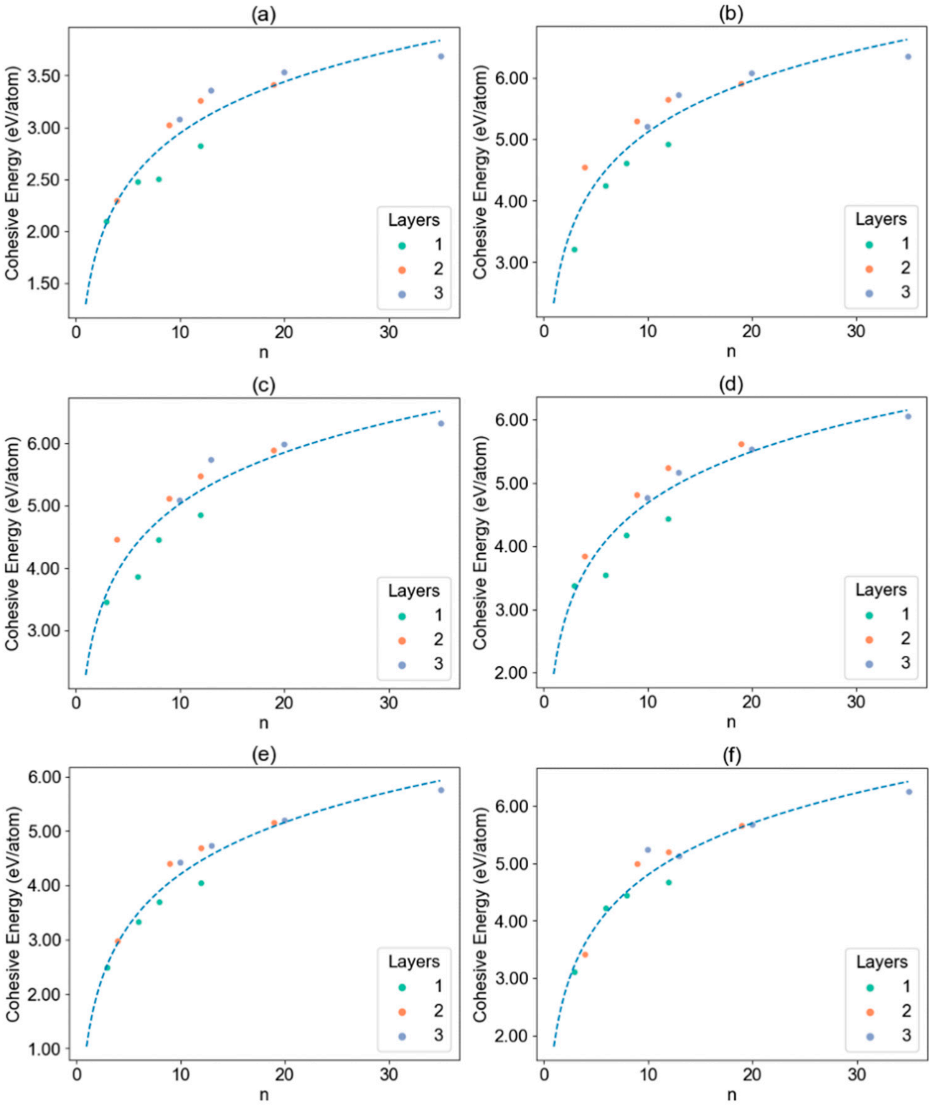

Figure S6: Calculated cohesive energy as a function of the nanocluster size (n) for transition-metal nanoclusters (X_n) in period 6 (a) X = La, (b) X = Hf, (c) X = Ta, (d) X = W, (e) X = Re, and (f) X = Os.

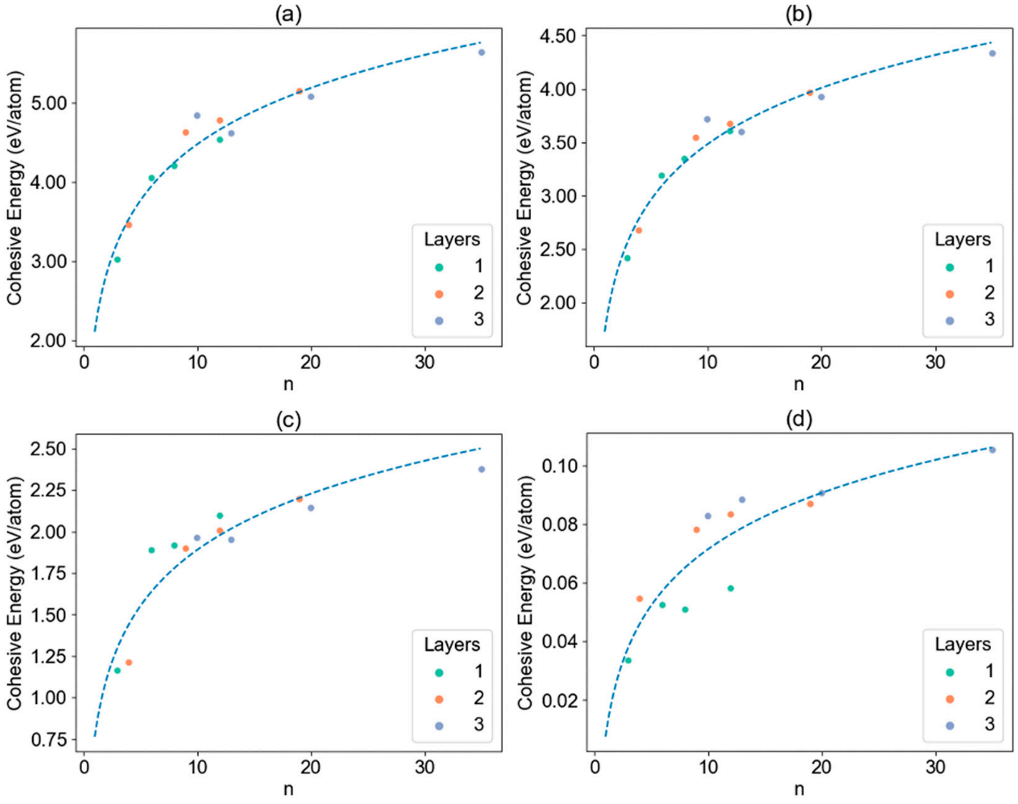

Figure S7: Calculated cohesive energy as a function of the nanocluster size (n) for transition-metal nanoclusters (X_n) in period 6 (a) X = Ir, (b) X = Pt, (c) X = Au, and (d) X = Hg.

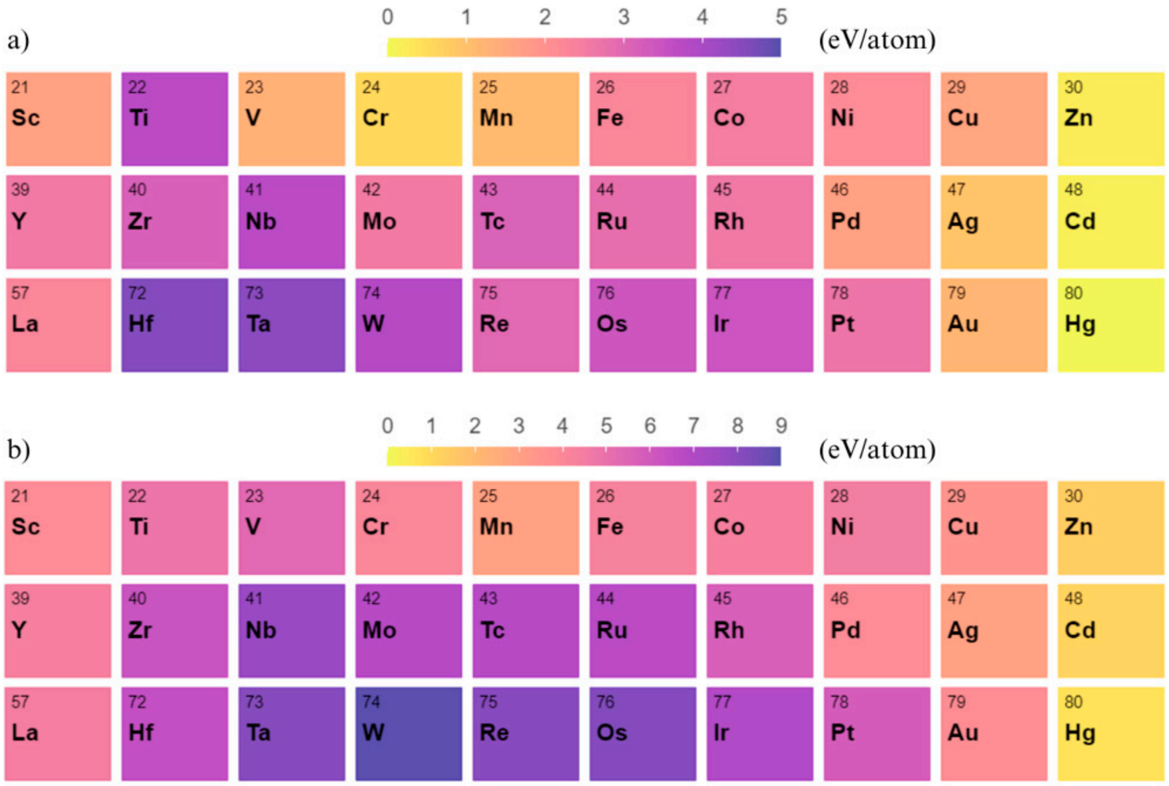

Figure S8: Heat map illustrating (a) the calculated cohesive energy (eV/atom) for transition-metal nanoclusters X_4 and (b) the experimental values of the transition-metal bulk structures. Experimental data sourced from Kittel, Charles. "*Introduction to Solid State Physics*", 8th edition. John Wiley & Sons, Inc., 2005.

Table S1: List of Parameters '*a*' and '*b*' in the Natural Logarithmic Function for Fitting Cohesive Energies of Nanoclusters.

Element	*a*	*b*
Sc	0.72504841	2.20934348
Ti	0.89810472	12.01789744
V	1.16951657	1.02238005
Cr	0.79397321	0.48902491
Mn	0.61779602	1.1861684
Fe	0.80001186	3.91109861
Co	0.824159	4.70718674
Ni	0.72964512	4.99065518
Cu	0.55160499	4.20072647
Zn	0.1913661	0.55464114
Y	0.75614744	5.3667668
Zr	1.00990119	4.09499696
Nb	1.04150976	6.20878157
Mo	1.35036784	0.96117769
Tc	1.11531218	3.74701285
Ru	1.01494177	4.02485467
Rh	0.83450127	5.01848205
Pd	0.58276816	3.03765054
Ag	0.42541707	3.13586064
Cd	0.11033387	0.67963352
La	0.71446756	6.1480644
Hf	1.21114911	6.83491705
Ta	1.18925133	6.83612842
W	1.17388636	5.396755
Re	1.37633963	2.12208248
Os	1.30260075	3.99292611
Ir	1.02817762	7.76355096
Pt	0.76148922	9.64927626
Au	0.48851975	4.77290778

Hg	0.02775863	1.30970181

Table S2: Calculated cohesive energy (E_{coh}) of the transition-metal nanoclusters (X_n) in the group 3B (X=Sc, Y, and La).

**Nanocluster**	**E_{coh} (eV/atom)**	**Nanocluster**	**E_{coh} (eV/atom)**	**Nanocluster**	**E_{coh} (eV/atom)**
Sc_3	1.24	Y_3	1.96	La_3	2.09
Sc_6	1.66	Y_6	2.44	La_6	2.47
Sc_8	1.97	Y_8	2.68	La_8	2.50
Sc_{12}	2.15	Y_{12}	2.86	La_{12}	2.82
Sc_{3-1}	1.65	Y_{3-1}	2.58	La_{3-1}	2.29
Sc_{6-3}	2.36	Y_{6-3}	3.10	La_{6-3}	3.02
Sc_{8-4}	2.57	Y_{8-4}	3.32	La_{8-4}	3.25
Sc_{12-7}	2.81	Y_{12-7}	3.54	La_{12-7}	3.41
Sc_{6-3-1}	2.34	Y_{6-3-1}	3.07	La_{6-3-1}	3.07
Sc_{3-7-3}	2.65	Y_{3-7-3}	3.33	La_{3-7-3}	3.35
Sc_{5-10-5}	2.91	Y_{5-10-5}	3.62	La_{5-10-5}	3.53
$Sc_{14-13-8}$	2.80	$Y_{14-13-8}$	3.84	$La_{14-13-8}$	3.68

Table S3: Calculated cohesive energy (E_{coh}) of the transition-metal nanoclusters (X_n) in the group 4B (X=Ti, Zr, and Hf).

**Nanocluster**	E_{coh} **(eV/atom)**	**Nanocluster**	E_{coh} **(eV/atom)**	**Nanocluster**	E_{coh} **(eV/atom)**
Ti_3	3.25	Zr_3	2.48	Hf_3	3.20
Ti_6	3.43	Zr_6	2.91	Hf_6	4.24
Ti_8	3.88	Zr_8	3.19	Hf_8	4.61
Ti_{12}	4.09	Zr_{12}	3.56	Hf_{12}	4.92
Ti_{3-1}	3.75	Zr_{3-1}	3.16	Hf_{3-1}	4.54
Ti_{6-3}	4.35	Zr_{6-3}	3.87	Hf_{6-3}	5.29
Ti_{8-4}	4.70	Zr_{8-4}	4.20	Hf_{8-4}	5.64
Ti_{12-7}	4.96	Zr_{12-7}	4.49	Hf_{12-7}	5.91
Ti_{6-3-1}	4.29	Zr_{6-3-1}	3.69	Hf_{6-3-1}	5.21
Ti_{3-7-3}	4.77	Zr_{3-7-3}	4.20	Hf_{3-7-3}	5.72
Ti_{5-10-5}	5.08	Zr_{5-10-5}	4.59	Hf_{5-10-5}	6.08
$Ti_{14-13-8}$	5.30	$Zr_{14-13-8}$	4.89	$Hf_{14-13-8}$	6.35

Table S4: Calculated cohesive energy (E_{coh}) of the transition-metal nanoclusters (X_n) in the group 5B (X=V, Nb, and Ta).

Nanocluster	E_{coh} (eV/atom)	Nanocluster	E_{coh} (eV/atom)	Nanocluster	E_{coh} (eV/atom)
V_3	1.17	Nb_3	2.73	Ta_3	3.45
V_6	2.23	Nb_6	3.48	Ta_6	3.86
V_8	2.53	Nb_8	3.91	Ta_8	4.45
V_{12}	2.73	Nb_{12}	4.11	Ta_{12}	4.84
V_{3-1}	1.33	Nb_{3-1}	3.76	Ta_{3-1}	4.45
V_{6-3}	2.85	Nb_{6-3}	4.39	Ta_{6-3}	5.11
V_{8-4}	3.24	Nb_{8-4}	4.75	Ta_{8-4}	5.47
V_{12-7}	3.50	Nb_{12-7}	5.03	Ta_{12-7}	5.88
V_{6-3-1}	2.87	Nb_{6-3-1}	4.38	Ta_{6-3-1}	5.08
V_{3-7-3}	3.10	Nb_{3-7-3}	4.92	Ta_{3-7-3}	5.73
V_{5-10-5}	3.51	Nb_{5-10-5}	4.97	Ta_{5-10-5}	5.98
$V_{14-13-8}$	3.86	$Nb_{14-13-8}$	5.43	$Ta_{14-13-8}$	6.31

Table S5: Calculated cohesive energy (E_{coh}) of the transition-metal nanoclusters (X_n) in the group 6B (X=Cr, Mo, and W).

Nanocluster	E_{coh} (eV/atom)	Nanocluster	E_{coh} (eV/atom)	Nanocluster	E_{coh} (eV/atom)
Cr_3	0.43	Mo_3	0.49	W_3	3.37
Cr_6	0.80	Mo_6	2.29	W_6	3.54
Cr_8	0.88	Mo_8	2.80	W_8	4.17
Cr_{12}	1.01	Mo_{12}	3.05	W_{12}	4.43
Cr_{3-1}	0.56	Mo_{3-1}	2.53	W_{3-1}	3.84
Cr_{6-3}	1.50	Mo_{6-3}	3.22	W_{6-3}	4.81
Cr_{8-4}	1.63	Mo_{8-4}	3.64	W_{8-4}	5.23
Cr_{12-7}	1.84	Mo_{12-7}	3.88	W_{12-7}	5.61
Cr_{6-3-1}	1.05	Mo_{6-3-1}	3.19	W_{6-3-1}	4.76
Cr_{3-7-3}	1.46	Mo_{3-7-3}	3.63	W_{3-7-3}	5.16
Cr_{5-10-5}	1.84	Mo_{5-10-5}	3.92	W_{5-10-5}	5.53
$Cr_{14-13-8}$	2.33	$Mo_{14-13-8}$	4.37	$W_{14-13-8}$	6.05

Table S6: Calculated cohesive energy (E_{coh}) of the transition-metal nanoclusters (X_n) in the group 7B (X=Mn, Tc, and Re).

**Nanocluster**	**E_{coh} (eV/atom)**	**Nanocluster**	**E_{coh} (eV/atom)**	**Nanocluster**	**E_{coh} (eV/atom)**
Mn_3	0.75	Tc_3	2.66	Re_3	2.48
Mn_6	1.06	Tc_6	3.31	Re_6	3.32
Mn_8	1.28	Tc_8	3.62	Re_8	3.69
Mn_{12}	1.43	Tc_{12}	3.85	Re_{12}	4.04
Mn_{3-1}	1.12	Tc_{3-1}	3.12	Re_{3-1}	2.97
Mn_{6-3}	1.53	Tc_{6-3}	4.14	Re_{6-3}	4.39
Mn_{8-4}	1.72	Tc_{8-4}	4.44	Re_{8-4}	4.68
Mn_{12-7}	1.94	Tc_{12-7}	4.82	Re_{12-7}	5.15
Mn_{6-3-1}	1.52	Tc_{6-3-1}	4.10	Re_{6-3-1}	4.42
Mn_{3-7-3}	1.88	Tc_{3-7-3}	4.52	Re_{3-7-3}	4.72
Mn_{5-10-5}	2.04	Tc_{5-10-5}	4.87	Re_{5-10-5}	5.19
$Mn_{14-13-8}$	2.22	$Tc_{14-13-8}$	5.32	$Re_{14-13-8}$	5.75

Table S7: Calculated cohesive energy (E_{coh}) of the transition-metal nanoclusters (X_n) in the group 8B (x=Fe, Ru, Os).

**Nanocluster**	**E_{coh} (eV/atom)**	**Nanocluster**	**E_{coh} (eV/atom)**	**Nanocluster**	**E_{coh} (eV/atom)**
Fe_3	1.89	Ru_3	2.57	Os_3	3.10
Fe_6	2.38	Ru_6	3.07	Os_6	4.22
Fe_8	2.65	Ru_8	3.31	Os_8	4.44
Fe_{12}	2.87	Ru_{12}	3.61	Os_{12}	4.67
Fe_{3-1}	2.29	Ru_{3-1}	2.83	Os_{3-1}	3.41
Fe_{6-3}	3.00	Ru_{6-3}	3.86	Os_{6-3}	4.99
Fe_{8-4}	3.22	Ru_{8-4}	4.07	Os_{8-4}	5.20
Fe_{12-7}	3.47	Ru_{12-7}	4.47	Os_{12-7}	5.66
Fe_{6-3-1}	2.98	Ru_{6-3-1}	3.98	Os_{6-3-1}	5.24
Fe_{3-7-3}	3.33	Ru_{3-7-3}	4.04	Os_{3-7-3}	5.13
Fe_{5-10-5}	3.54	Ru_{5-10-5}	4.48	Os_{5-10-5}	5.68
$Fe_{14-13-8}$	3.78	$Ru_{14-13-8}$	4.97	$Os_{14-13-8}$	6.25

Table S8: Calculated cohesive energy (E_{coh}) of the transition-metal nanoclusters (X_n) in the group 8B (X=Co, Rh, and Ir).

Nanocluster	E_{coh} (eV/atom)	Nanocluster	E_{coh} (eV/atom)	Nanocluster	E_{coh} (eV/atom)
Co_3	2.02	Rh_3	2.17	Ir_3	3.02
Co_6	2.75	Rh_6	2.72	Ir_6	4.05
Co_8	2.88	Rh_8	2.99	Ir_8	4.20
Co_{12}	3.13	Rh_{12}	3.18	Ir_{12}	4.53
Co_{3-1}	2.49	Rh_{3-1}	2.60	Ir_{3-1}	3.46
Co_{6-3}	3.28	Rh_{6-3}	3.31	Ir_{6-3}	4.62
Co_{8-4}	3.43	Rh_{8-4}	3.54	Ir_{8-4}	4.78
Co_{12-7}	3.71	Rh_{12-7}	3.83	Ir_{12-7}	5.15
Co_{6-3-1}	3.32	Rh_{6-3-1}	3.44	Ir_{6-3-1}	4.84
Co_{3-7-3}	3.46	Rh_{3-7-3}	3.57	Ir_{3-7-3}	4.61
Co_{5-10-5}	3.77	Rh_{5-10-5}	3.86	Ir_{5-10-5}	5.07
$Co_{14-13-8}$	4.08	$Rh_{14-13-8}$	4.22	$Ir_{14-13-8}$	5.64

Table S9: Calculated cohesive energy (E_{coh}) of the transition-metal nanoclusters (X_n) in the group 8B (X = Ni, Pd, and Pt).

**Nanocluster**	E_{coh} **(eV/atom)**	**Nanocluster**	E_{coh} **(eV/atom)**	**Nanocluster**	E_{coh} **(eV/atom)**
Ni_3	1.86	Pd_3	1.23	Pt_3	2.41
Ni_6	2.48	Pd_6	1.52	Pt_6	3.19
Ni_8	2.61	Pd_8	1.73	Pt_8	3.34
Ni_{12}	2.84	Pd_{12}	1.94	Pt_{12}	3.60
Ni_{3-1}	2.16	Pd_{3-1}	1.64	Pt_{3-1}	2.67
Ni_{6-3}	2.94	Pd_{6-3}	2.01	Pt_{6-3}	3.54
Ni_{8-4}	3.12	Pd_{8-4}	2.18	Pt_{8-4}	3.67
Ni_{12-7}	3.32	Pd_{12-7}	2.38	Pt_{12-7}	3.96
Ni_{6-3-1}	3.05	Pd_{6-3-1}	2.06	Pt_{6-3-1}	3.71
Ni_{3-7-3}	3.06	Pd_{3-7-3}	2.23	Pt_{3-7-3}	3.59
Ni_{5-10-5}	3.34	Pd_{5-10-5}	2.45	Pt_{5-10-5}	3.92
$Ni_{14-13-8}$	3.65	$Pd_{14-13-8}$	2.67	$Pt_{14-13-8}$	4.33

Table S10: Calculated cohesive energy (E_{coh}) of the transition-metal nanoclusters (X_n) in the group 1B (X=Cu, Ag, and Au).

Nanocluster	E_{coh} (eV/atom)	Nanocluster	E_{coh} (eV/atom)	Nanocluster	E_{coh} (eV/atom)
Cu_3	1.21	Ag_3	0.87	Au_3	1.16
Cu_6	1.90	Ag_6	1.42	Au_6	1.89
Cu_8	1.97	Ag_8	1.46	Au_8	1.91
Cu_{12}	2.16	Ag_{12}	1.60	Au_{12}	2.09
Cu_{3-1}	1.57	Ag_{3-1}	0.90	Au_{3-1}	1.21
Cu_{6-3}	2.10	Ag_{6-3}	1.50	Au_{6-3}	1.90
Cu_{8-4}	2.21	Ag_{8-4}	1.58	Au_{8-4}	2.00
Cu_{12-7}	2.41	Ag_{12-7}	1.73	Au_{12-7}	2.19
Cu_{6-3-1}	2.08	Ag_{6-3-1}	1.47	Au_{6-3-1}	1.96
Cu_{3-7-3}	2.20	Ag_{3-7-3}	1.56	Au_{3-7-3}	1.95
Cu_{5-10-5}	2.43	Ag_{5-10-5}	1.74	Au_{5-10-5}	2.14
$Cu_{14-13-8}$	2.65	$Ag_{14-13-8}$	1.89	$Au_{14-13-8}$	2.37

Table S11: Calculated cohesive energy (E_{coh}) of the transition-metal nanoclusters (X_n) in the group 2B (X=Zn, Cd, and Hg).

Nanocluster	E_{coh} (eV/atom)	Nanocluster	E_{coh} (eV/atom)	Nanocluster	E_{coh} (eV/atom)
Zn_3	0.10	Cd_3	0.08	Hg_3	0.03
Zn_6	0.18	Cd_6	0.13	Hg_6	0.05
Zn_8	0.20	Cd_8	0.14	Hg_8	0.05
Zn_{12}	0.22	Cd_{12}	0.16	Hg_{12}	0.06
Zn_{3-1}	0.24	Cd_{3-1}	0.16	Hg_{3-1}	0.05
Zn_{6-3}	0.34	Cd_{6-3}	0.23	Hg_{6-3}	0.08
Zn_{8-4}	0.41	Cd_{8-4}	0.26	Hg_{8-4}	0.08
Zn_{12-7}	0.49	Cd_{12-7}	0.28	Hg_{12-7}	0.09
Zn_{6-3-1}	0.41	Cd_{6-3-1}	0.26	Hg_{6-3-1}	0.08
Zn_{3-7-3}	0.34	Cd_{3-7-3}	0.21	Hg_{3-7-3}	0.09
Zn_{5-10-5}	0.41	Cd_{5-10-5}	0.27	Hg_{5-10-5}	0.09
$Zn_{14-13-8}$	0.63	$Cd_{14-13-8}$	0.39	$Hg_{14-13-8}$	0.11
